# Antioxidant, Metal Chelating, Anti-glucosidase Activities and Phytochemical Analysis of Selected Tropical Medicinal Plants

**Published:** 2014

**Authors:** Fai-Chu Wong, Ann-Li Yong, Evon Peir-Shan Ting, Sim-Chyi Khoo, Hean-Chooi Ong, Tsun-Thai Chai

**Affiliations:** a *Centre for Biodiversity Research, Universiti Tunku Abdul Rahman, 31900 Kampar, Malaysia.*; b *Department of Chemical Science, Faculty of Science, Universiti Tunku Abdul Rahman, 31900 Kampar, Malaysia.*; c *Institute of Biological Sciences, Faculty of Science, University of Malaya, 50603 Kuala Lumpur, Malaysia.*

**Keywords:** Antioxidation, Anti-glucosidase, Caffeic acid, Diabetes, Gallic acid, Phenolic compounds, Quercetin

## Abstract

*The purpose of this investigation was to determine the antioxidant potentials and anti-glucosidase* activities of six tropical medicinal plants. The levels of phenolic constituents in these medicinal plants were also quantified and compared. *Antioxidation potentials were determined colorimetrically for scavenging activities against DPPH and NO radicals. Metal chelating assay was based on the measurement of iron-ferrozine absorbance at 562 nm. Anti-diabetic potentials were measured by using α-glucosidase as target enzyme. Medicinal plants’ total phenolic, total flavonoid and hydroxycinnamic acid contents were determined using spectrophotometric methods, *by comparison to standard plots prepared using gallic acid, quercetin and caffeic acid standards, respectively. Radical scavenging and metal chelating activities were detected in all medicinal plants, in concentration-dependent manners. Among the six plants tested, *C. nutans*, *C. formosana* and *H. diffusa* were found to possess α-glucosidase inhibitory activities. Spectrophotometric analysis indicated that the total phenolic, total flavonoid and hydroxycinnamic acid contents ranged from 12.13-21.39 mg GAE per g of dry sample, 1.83-9.86 mg QE per g of dry sample, and 0.91-2.74 mg CAE per g of dry sample, respectively. Our results suggested that *C. nutans* and *C. formosana* could potentially be used for the isolation of potent antioxidants and anti-diabetic compounds. To the best of our knowledge, this study represents the first time that *C. nutans* (Acanthaceae family) was reported in literature with glucosidase inhibition activity.

## Introduction

In the normal aging process, oxidative stress accumulates in our bodies and leads to the onset of various diseases. Oxidative stress is frequently linked to the occurrences of excess free radicals such as reactive oxygen and nitrogen species, which could be generated during the body normal metabolic activities and from outer environmental factors, such as uv radiation and chemical pollutants (1). Scientific studies have demonstrated the direct relationships between excess free radicals, DNA damages and cell malignancy. In addition, oxidative stress is implicated as one of the main factors responsible for the induction of type II diabetes mellitus (2, 3). The healthcare costs of these chronic diseases are enormous, and solutions are urgently needed to ease the burdens of individuals and society. 

Medicinal plants, rich with their secondary metabolites, offer a reservoir of preventive and therapeutic options. Through the efforts of ongoing scientific researches, increasing number of phytochemicals have been tested and developed into effective modern drugs. Examples of these plant-derived therapeutic agents include vinblastine and vincristine, two alkaloid drugs widely used in chemotherapy, which are derived originally from *Catharanthus roseus* (Madagascar periwinkle) (4). An additional example is etoposide, a non-alkaloid anticancer drug derived from the North American *Podophyllum* species (Mayapple) (5). An added benefit of plant-derived bioactive compounds is the minimal side effect, compared to those of synthetic drugs. It is hoped that with more researches focusing on medicinal plants, effective therapeutic agents with minimal side effects could be identified. 

In this paper, we aimed to quantify and compare the antioxidation and anti-diabetic potentials of six selected tropical medicinal plants, namely *Callicarpa formosana*, *Clinacanthus nutans*, *Hedyotis diffusa*, *Leonurus cardiaca*, *Pereskia bleo* and *Vernonia* *amygdalina*. These medicinal plants are frequently consumed by the local communities in the forms of health-promoting tonics and herbal drinks. The radical scavenging potentials were accessed using two different methods, namely DPPH and nitric oxide (NO) radical scavenging assays. Metal chelating activities were also determined, as excess free metals have been implicated in the induction and formation of free radicals (6). For the testing of anti-diabetic potentials, we used α-glucosidase as the inhibition target. Additionally, phytochemical levels of these medicinal plants were also determined. Through this study, we aimed to identify medicinal plants with potential preventative values against cancer, diabetes and other oxidative-induced diseases. 

## Experimental

*Preparation of herbal extracts*

Medicinal plants were collected from March to June of 2012. The plant species were authenticated by Professor Dr. Ong Hean Chooi at the Institute of Biological Sciences, University of Malaya, Malaysia. Voucher specimens (Voucher Numbers: MHR-2012-006 to MHR-2012-011) were deposited at Faculty of Science, Universiti Tunku Abdul Rahman. The medicinal plants were dried in an oven at 40 ºC for 48 h or until constant weight was observed. Each dried plant sample was then pulverized. Plant samples were then incubated with distilled water at 1:19 (w/v), followed by heating at 90 ºC for an hour. Supernatant was then filtered using cheesecloth and centrifuged at 10,000 rpm for 10 min. Clarified medicinal plant extracts were then aliquoted and stored at -20 ºC until testing.

*Determination of 1,1-diphenyl-2-picrylhydrazyl* *(DPPH)* *radical scavenging activity*

DPPH radical scavenging activity was assessed as described previously with modifications (7). Briefly, to 1 mL of DPPH working solution, 50 µL of extract was added. The mixture was left in the dark for 30 min before its absorbance was read at 517 nm. DPPH radical scavenging activity was calculated using the following formula: 

DPPH radical scavenging activity = (A_control _– A_sample_)/A_control_ x 100

Where A_control_ is the absorbance of control reaction (without plant extract), and A_sample _is the absorbance in the presence of a plant extract. 

*Determination of nitric oxide* *(NO)* *radical scavenging activity*

NO radical scavenging assay was performed as described (7). Briefly, 0.8 mL of plant extract was added into 0.2 mL of freshly prepared sodium nitroprusside (5 mM, in phosphate buffer saline, pH 7.4). The mixture was incubated at room temperature under light source (24 W compact fluorescent light bulb). After 150 min, 0.6 mL of the mixture was transferred into a new tube containing 0.6 mL of Griess Reagent (1% sulphanilamide and 0.1% N-(1-naphthyl)-ethyleneadiamine dihydrochloride in 5% phosphoric acid). After incubating for 10 min in darkness, absorbance was recorded at 546 nm. NO radical scavenging activity was calculated using a formula similar to that used for DPPH radical scavenging assay.

*Determination of metal chelating activity*

Metal chelating activity was measured as described previously, by adding 0.1 mM FeSO_4 _(0.2 mL) and 0.25 mM ferrozine (0.4 mL) subsequently into 0.2 mL of plant extract (8). After incubating at room temperature for 10 min, absorbance of the mixture was recorded at 562 nm. Chelating activity was calculated using the following formula: 

Metal chelating activity = (A_control _– A_sample_)/A_control_ x 100

Where A_control_ is the absorbance of control reaction (without plant extract), and A_sample _is the absorbance in the presence of a plant extract.

*Alpha-glucosidase inhibition assay*

Glucosidase inhibition activity was measured as previously described (9) by mixing the following components in sequential order: 250 µL of 100 mM potassium phosphate buffer (pH 7.0), 150 µL of 0.5 mM 4-nitrophenyl-α-D-glucopyranoside, 50 µL plant extracts, and 150 µL of α-glucosidase (0.1 unit/mL in 10 mM potassium phosphate buffer). After incubating at 37 ºC for 30 min, reaction was stopped by the addition of 600 µL of 200 mM Na_2_CO_3_, and the absorbance was recorded at 400 nm. Glucosidase inhibition activity was calculated using a formula similar to that used for metal chelating assay.

*Determination of phytochemical contents*

Phytochemical contents (total phenolics, total flavonoids and hydroxycinnamic acids) were determined as previously described (7, 10). Briefly, total phenolic content was determined using Folin-Ciocalteu colorimetric assay, and result was reported as mg gallic equivalents/g dry matter (11). Total flavonoid content was determined using NaNO_2_ and AlCl_3_.6H_2_O, and result was expressed as mg quercetin equivalents/g DM. Hydroxycinnamic acid content was determined using Arnow’s reagent, and absorbance was recorded at 490 nm. The total hydroxycinnamic acid content was then determined from a caffeic acid standard curve, and result was expressed as mg caffeic acid equivalent/g DM.

*Statistical analysis*

Data were reported as mean standard errors, obtained from three replicate trials. Statistical analysis was performed using SAS (Version 9.2). Data were analyzed using ANOVA test, and means of significant differences were separated using Fisher’s Least Significant Difference test (at the 0.05 level of probability). 

## Results

*DPPH and NO radical scavenging activities*

**Figure 1:**
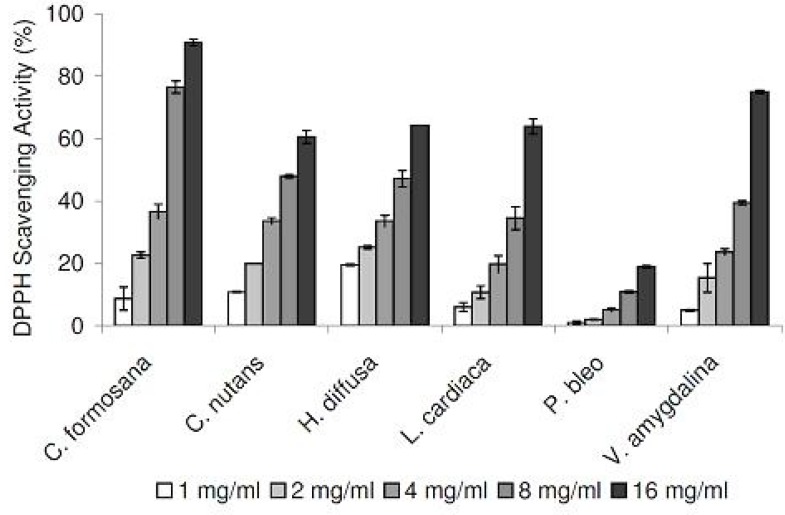
DPPH radical scavenging activities of plant extracts at different concentrations. Data are reported as mean ± SE values (n=3).

**Figure 2:**
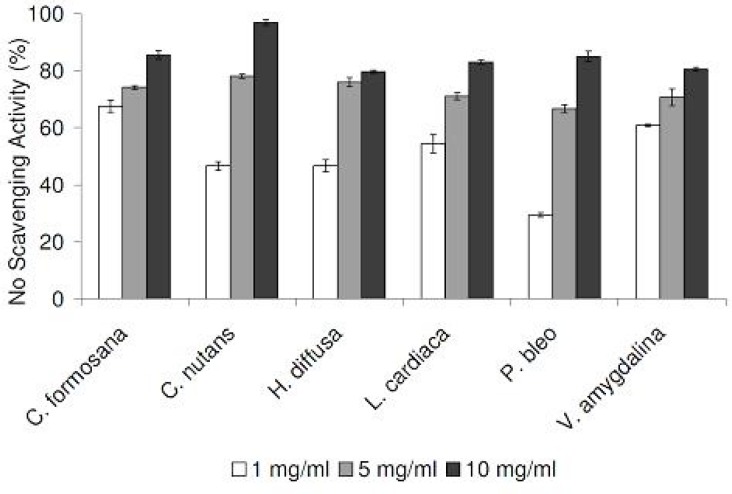
NO radical scavenging activities of plant extracts at different concentrations. Data are reported as mean ± SE values (n=3).

In our DPPH radical scavenging assay, all plant extracts exhibited scavenging activities in a concentration-dependent manner, in the range of 1 to 16 mg/mL (Figure 1). Compared at the concentration of 16 mg/mL, *C. formosana* demonstrated the highest DPPH scavenging activity, while the lowest activity was detected in *P. bleo*. In addition, we tested our plant extracts in a NO radical scavenging assay. As NO radicals are found in biological systems, the results could be more biologically relevant. NO radical scavenging activities were detected in all plant extracts, in a concentration-dependent manner (Figure 2). Despite its low DPPH radical scavenging activity, *P. bleo* demonstrated strong NO radical scavenging activity, which was comparable to that of *C. formosana* and other plant extracts. 

*Metal chelating activities*

**Figure 3:**
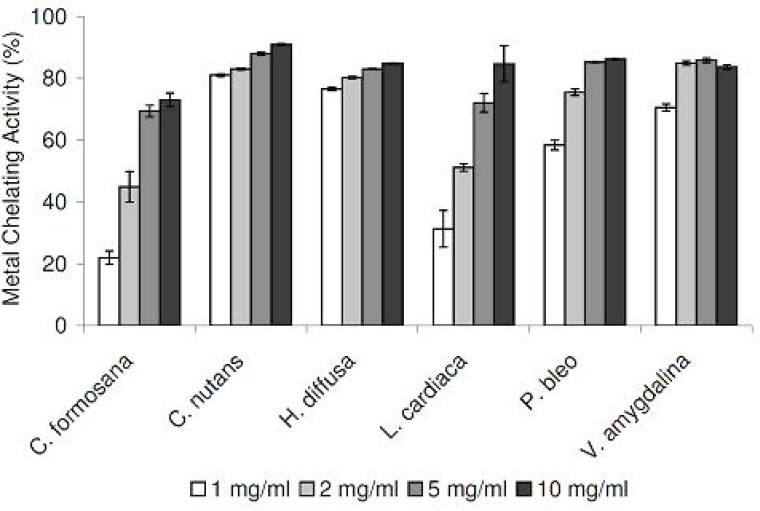
Metal chelating activities of plant extracts at different concentrations. Data are reported as mean ± SE values (n=3).

As excess free irons have been implicated in the induction and formation of free radicals in biological systems, we tested our medicinal plant extracts in a metal chelating assay. Tested in the concentration range of 1 to 10 mg/mL, all six extracts demonstrated strong chelating activities in concentration-dependent manners (Figure 3). Compared at the concentration of 1 mg/mL, *C. nutans* and *H. diffusa* demonstrated the strongest activities, while the weakest activities were detected in *C. formosana* and *L. cardiaca*.

*α-glucosidase inhibition activities*

**Figure 4:**
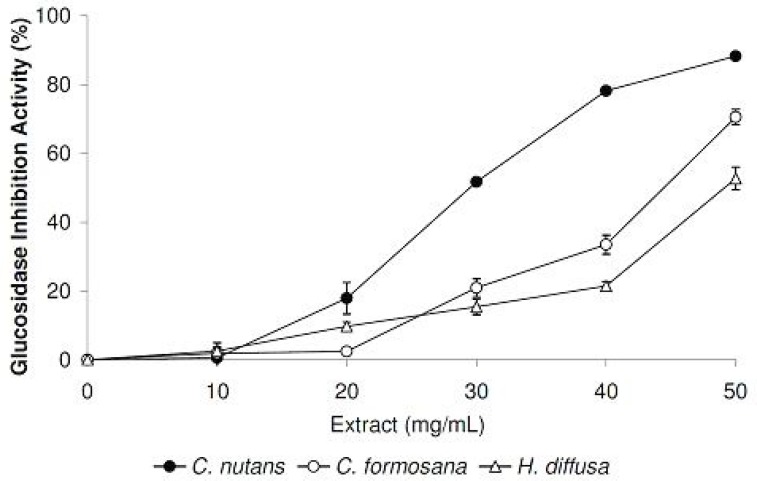
Glucosidase inhibitory activities of plant extracts at different concentrations. Data are reported as mean ± SE values (n=3).

The six medicinal plant extracts were also tested for their potential therapeutic applications to treat type II diabetes mellitus. In this effort, α-glucosidase was chosen as the target enzyme, as it is involved in converting starch into absorbable monosaccharides. In our initial screening at the concentration of 50 mg/mL, α-glucosidase inhibitory activities (%) were detected in the following order: *C. nutans* (88.2 ± 0.4) > *C. formosana* (70.5 ± 2.3) > *H. diffusa* (52.7 ± 3.3) > *V. amygdalina* (18.9 ± 2.6) > *P. bleo* (12.1 ± 1.8). For plants extracts with inhibitory activities larger than 50%, we also performed a detailed α-glucosidase inhibition assay using different extract concentrations (Figure 4).

*Total phenolic, total flavonoid and hydroxycinnamic acid contents*

As the bioactivities of medicinal plant extracts were often linked to their phytochemical contents, we also determined the total phenolic, total flavonoid and hydroxycinnamic acid contents of our medicinal plant extracts. The highest total phenolic contents were detected in *H. diffusa* and *V. amygdalina* (>20 mg GAE/g dry matter), followed by *C. formosana*, and the group of *C. nutans*, *P. bleo* and *L. cardiaca* (<15 mg GAE/g dry matter)(Table 1). Similar trend was observed in the hydroxycinnamic acid contents, with the highest levels detected in *V. amygdalina* and *H. diffusa*, and the lowest level detected in *C. nutans*. Interestingly, the highest level of total flavonoids was detected in *L. cardiaca* (9.86 mg QE/g dry matter), despite its low total phenolic content, while the lowest level of total flavonoids was detected in *C. nutans* (2.08 mg QE/g dry matter).

**Table 1:** Total phenolic, total flavonoid and hydroxycinnamic acid contents of the six medicinal herbs.

**Herbs**	**Total phenolics** **(mg GAE/g DM)** ^(1)^	**Total flavonoids** **(mg QE/g DM)** ^(2)^	**Hydroxycinnamic acids** **(mg CAE/g DM)** ^(3),(4)^
***C. formosana***	18.17 ± 0.10^c^	8.77 ± 0.99^b^	1.63 ± 0.14^c^
***C. nutans***	14.70 ± 0.08^d^	2.07 ± 0.05^e^	0.91 ± 0.01^d^
***H. diffusa***	21.39 ± 0.36^a^	6.30 ± 0.10^c^	1.98 ± 0.01^b^
***L. cardiaca***	12.13 ± 0.12^e^	9.86 ± 0.15^a^	2.01 ± 0.01^b^
***P. bleo***	14.16 ± 0.15^d^	1.83 ± 0.07^e^	1.52 ± 0.01^c^
***V. amygdalina***	20.22 ± 0.03^b^	4.82 ± 0.01^d^	2.74 ± 0.01^a^

## Discussion

To assess the antioxidation capabilities of these medicinal plant extracts, we resorted to use two different radical scavenging assays. In addition to DPPH radicals, plant extracts were also tested for their abilities to scavenge NO radicals. The latter was included as it is believed to be more biologically relevant, since NO radicals are present in our bodies (12). Plant extracts were also assessed for their metal chelating activities, which are linked to antioxidation capabilities. All medicinal plants tested exhibited DPPH and NO scavenging activities, in concentration-dependent manners (Figure 1 and 2). In the DPPH radical scavenging assay, we observed that *C. formosana* demonstrated the highest radical scavenging activity, while the lowest activity was detected in *P. bleo*. Interestingly, despite its low DPPH scavenging potential, *P. bleo* demonstrated strong NO radical scavenging potential, as well as strong metal chelating potential, comparable to those of *C. formosana*. This observation highlighted the necessity to include different radical scavenging assays, during the assessment of medicinal plants’ antioxidation potentials.

Recently, *C. nutans* received a lot of scientific attentions, as it was reported with potent antiproliferative effects on different cancer cell lines (13). In our analyses, *C. nutans* was consistently found to exhibit strong antioxidation potentials, with high radical scavenging and metal chelating activities. It still remained unclear which chemical constituents were responsible for the potent antiproliferation and antioxidation activities. Compared to the other medicinal plants tested in this study, high level of total phenolic content was detected in *C. nutans*, while its levels of total flavonoid and hydroxycinnamic acid contents were among the lowest (Table 1). It is tempting to speculate that the observed antioxidative potential of *C. nutans* is contributed mainly by active constituents of the phenolic classes. Yet, detailed future studies are required to determine whether this is the case or not.

One of the main causes of type II diabetes mellitus is linked to the damage of pancreatic β-cells, which are induced by excess free radicals and the resultant oxidative stress (2). In our screening for potential glucosidase inhibitors, three of the medicinal plants tested were identified with promising glucosidase inhibition activities, namely *C*. *nutans*, *C. formosana* and *H. diffusa*. The glucosidase inhibition was especially noticeable with *C. nutans* extracts, which exhibited an 88% inhibition activity at a concentration of 50 mg/mL (Figure 4). Despite their significantly higher total phenolics and flavonoids levels, *C. formosana* and *H. diffusa* demonstrated lower glucosidase inhibition activities compared to *C. nutans*. This observation suggested that other phytochemical groups (alkaloids, terpenoids, *etc*.) may contribute to the glucosidase inhibition activity observed with *C. nutans*. To the best of our knowledge, this study represents the first time that *C. nutans* (Acanthaceae family) was reported in literature with glucosidase inhibition activity. 

Coincidentally, *Barleria lupulina*, another member of the same Acanthaceae family, was reported being used traditionally for diabetes treatments and showed anti-diabetic activities in study using animal models (14). These observations highlighted the need to test members of the Acanthaceae family for glucosidase inhibitory potentials. We also attempted to find a correlation between glucosidase inhibition activities with the phytochemical contents. However, no significant direct relationship was observed with the three groups of phytochemical tested in this study. It is possible that the glucosidase inhibition activity observed with *C. nutans* is contributed by other phytochemicals not tested in this study. Possible phytochemical include arjunolic acid, a triterpenoid saponin, which has previously been implicated with glucosidase inhibition activities (15). 

## Conclusion

We reported here the antioxidation and anti-diabetic potentials of six tropical medicinal plants. Radical scavenging and metal chelating activities were detected in all medicinal plants tested, in concentration dependent manners. The α-glucosidase inhibition assay revealed three medicinal plants with anti-diabetic potentials, with *C. nutans* showing the strongest inhibitory activity. Further study in this direction could potentially lead to the discovery of phytochemicals with promising antioxidation and anti-diabetic therapeutic values.
